# Publisher Correction: Misspecified models create the appearance of adaptive control during value-based choice

**DOI:** 10.1038/s44271-026-00419-6

**Published:** 2026-02-23

**Authors:** Harrison Ritz, Romy Frömer, Amitai Shenhav

**Affiliations:** 1 Cognitive, Linguistic, and Psychological Sciences, Brown University, Providence, RI USA; 2 Carney Institute for Brain Sciences, Brown University, Providence, RI USA; 3 Princeton Neuroscience Institute, Princeton University, Princeton, NJ USA; 4 School of Psychology, University of Birmingham, Birmingham, UK; 5 Centre for Human Brain Health, University of Birmingham, Birmingham, UK; 6 Department of Psychology, University of California, Berkeley, CA USA; 7 Helen Wills Neuroscience Institute, University of California, Berkeley, CA USA

**Keywords:** Human behaviour, Economics, Human behaviour

Correction to: *Communications Psychology* 10.1038/s44271-025-00374-8, published online 14 January 2026

In the PDF version of the article initially published, there was an error in the last term of Equation (1), where “– ∥∅ ∥” was originally rendered as “– ∅”; the equation is now amended in the PDF version of the article.

