# Supplementary material for: Misspecified models create the appearance of adaptive control during value-based choice
Source: Commun Psychol. 2026 Jan 14;4:11. doi: 10.1038/s44271-025-00374-8 (PMC12824220; doi:10.1038/s44271-025-00374-8)
Supplement: Supplementary file 2 — Supplementary Information [file 44271_2025_374_MOESM2_ESM.pdf]

Supplementary Figures

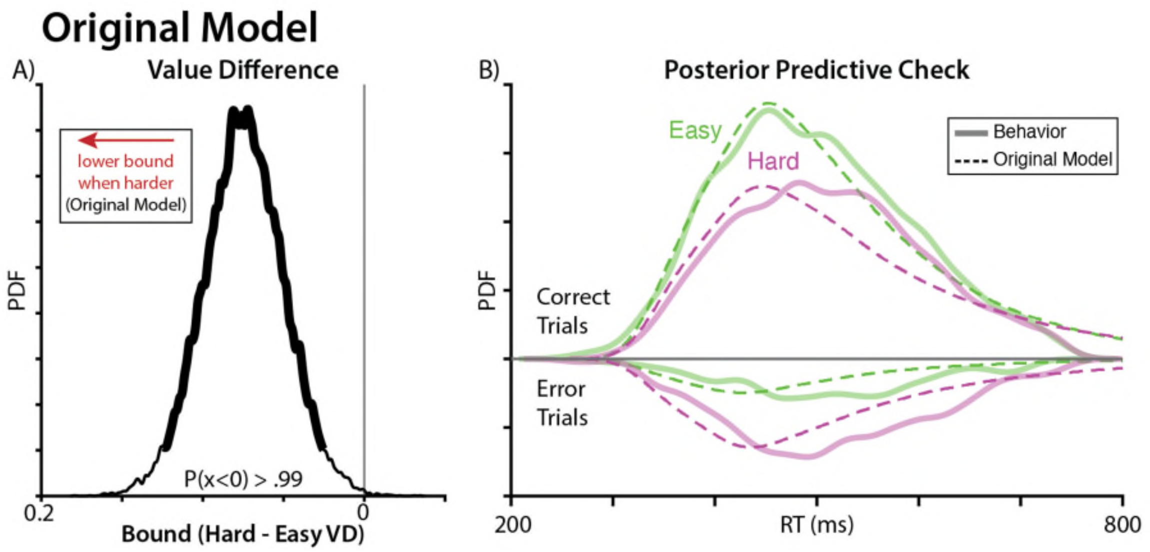

Supplementary Figure 1. Direct replication of analyses and findings in the original paper. A) When fitting a model with the exact specifications used by Vassena et al., we replicate their finding of harder choices being associated with lower decision thresholds. **B)** Predicted choice behavior from this original model provides a poor qualitative fit to observed accuracy and reaction time distributions. See Figure 1C-D for a version of this model aimed at improving fit without altering the model's core assumptions.

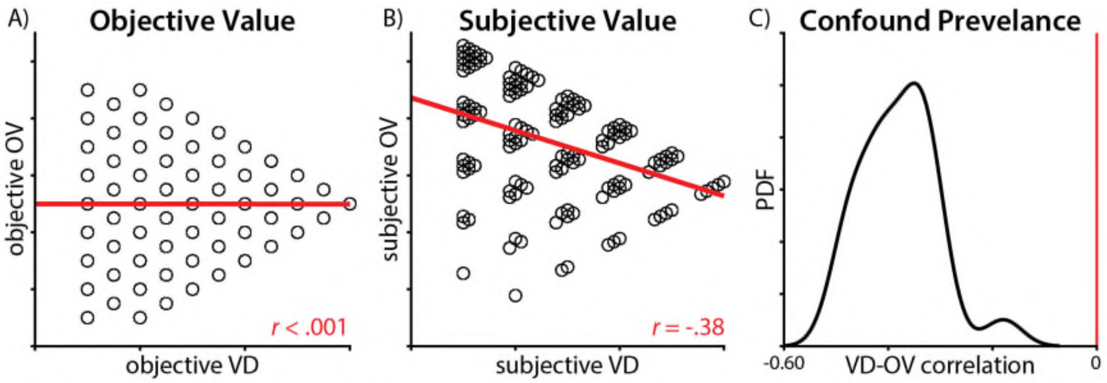

Supplementary Figure 2. Unequal weighting introduces confounding between overall value and value difference. A) If all outcomes were weighted equally (as in the Original and Original* models), value difference and overall value were uncorrelated by design. **B)** If participants have unequal weighting on the best and worst outcomes in each option (which improves model fit), value difference and overall value can become correlated. Here, we used the group-level drift rate weights estimated in the best-fitting model. **C)** Using the participant-specific drift rate weights, we found that all participants show some degree of correlation between overall value and value difference, introducing the potential for confounding.

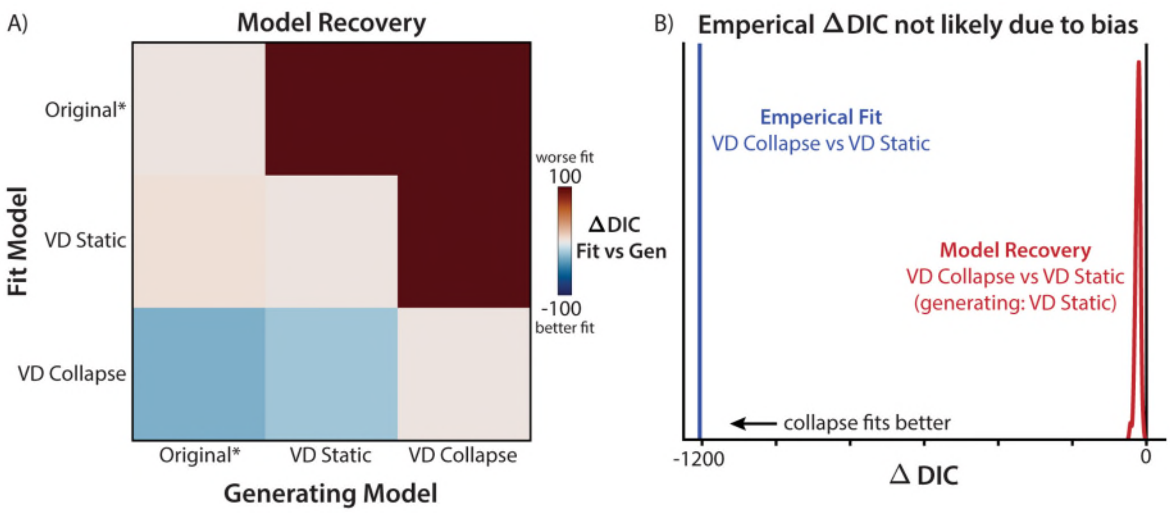

Supplementary Figure 3. Model Recovery. A) We simulated forty full datasets using parameters sampled from the posterior distribution from each generating model. Each model was then fitted (rows) to each generating dataset (columns). We computed the difference in model fit (DIC) between each model relative to the ground-truth model (diagonal). In the upper triangle, we find that more complex models were not confused for more simple models. In the lower triangle, whereas we found good recovery within static-bound models, the collapsing-bound model tended to fit better than the ground-truth model (lower row), indicating a bias. We fit both static- and collapsing-bound models using HDDM's likelihood approximation networks to match the likelihoods as closely as possible. We found similar results when using the standard HDDM procedure for static-bound models, which was the approach used in the results. **B)** When comparing VD Static and VD Collapse models, the bias observed in model recovery (see panel A, bottom row) was much smaller than the empirical model comparison. This suggests that empirical differences in model fit are unlikely to be due to biases in our model fitting and comparison procedure.This suggests that empirical differences in model fit are unlikely to be due to bias.

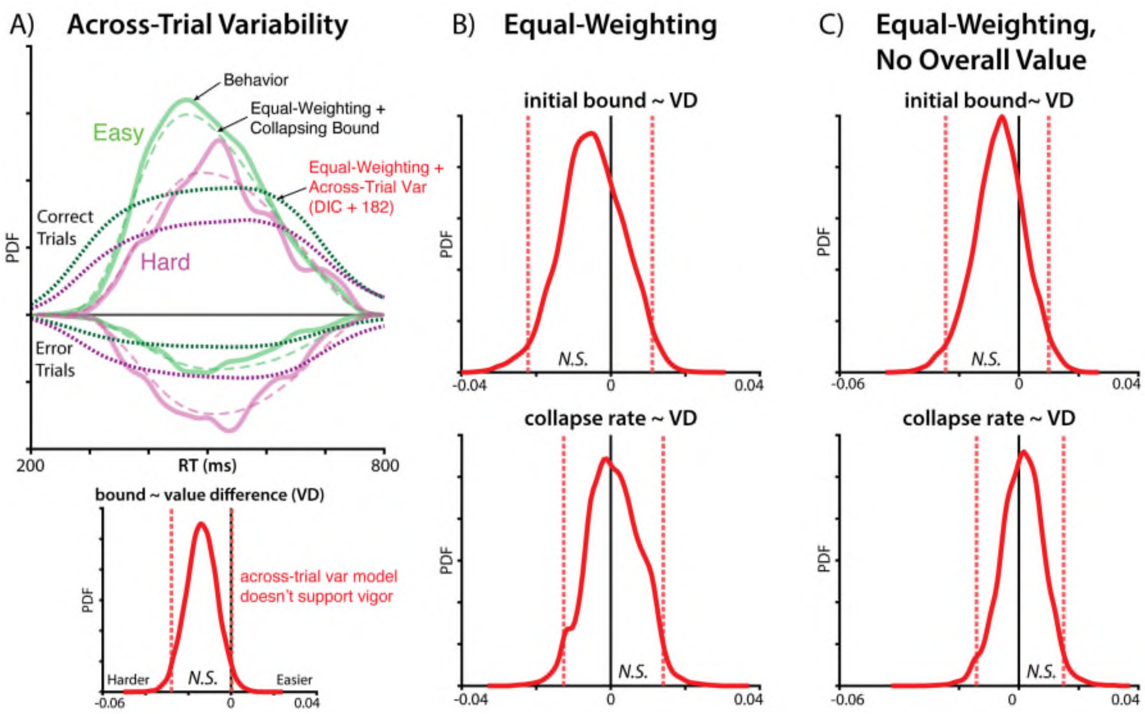

Supplementary Figure 4. Alternative models don't support vigor. A) Top: Posterior predictive checks for collapsing-bound models (long dash; as in the main manuscript) and fixed-bound models with across-trial variability in the threshold (short dash; poorer fit). Bottom: 95% credible intervals on the value-bound effect in the across-trial variability model overlap with zero. **B)** Models that equally weigh the maximum- and minimum-value option within each bundle do not find a credible value-bound effect. **C)** Models that equally weigh options and exclude overall value do not find a credible value-bound effect.

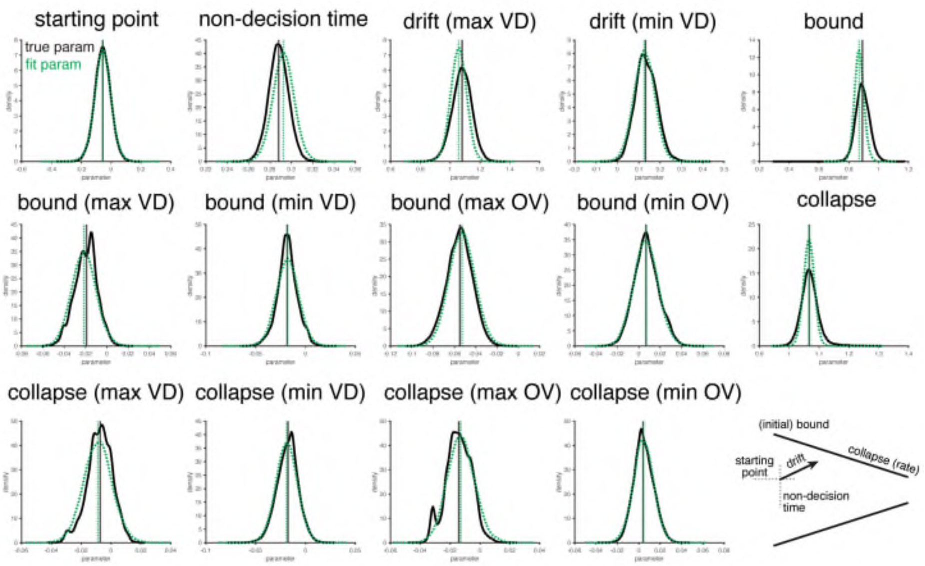

Supplementary Figure 5. Parameter Recovery. We simulated forty full datasets using parameters sampled from the posterior distributions of the best-fitting model. When we then fit the ground-truth model to the simulated datasets, we found good correspondence between the generating parameters (black, 'true') and the estimated parameters (green, 'fit'), suggesting that the parameters in our best-fitting model are identifiable. Note that between-simulation differences were subtracted from our estimated parameter, to remove variability due to factors like dataset sampling and the initialization of our fitting procedure.
