# Supplementary material for: Misspecified models create the appearance of adaptive control during value-based choice
Source: Commun Psychol. 2026 Jan 14;4:11. doi: 10.1038/s44271-025-00374-8 (PMC12824220; doi:10.1038/s44271-025-00374-8)
Supplement: Supplementary file 3 — Reporting Summary [file 44271_2025_374_MOESM3_ESM.pdf]

Corresponding author(s): Harrison Ritz

Last updated by author(s): Oct 29, 2025

Reporting Summary

Nature Portfolio wishes to improve the reproducibility of the work that we publish. This form provides structure for consistency and transparency in reporting. For further information on Nature Portfolio policies, see our Editorial Policies and the Editorial Policy Checklist.

Statistics

For all statistical analyses, confirm that the following items are present in the figure legend, table legend, main text, or Methods section.

n/a	Confirmed	
☐	☒	The exact sample size (n) for each experimental group/condition, given as a discrete number and unit of measurement
☐	☒	A statement on whether measurements were taken from distinct samples or whether the same sample was measured repeatedly
☐	☒	The statistical test(s) used AND whether they are one- or two-sided<br>*Only common tests should be described solely by name; describe more complex techniques in the Methods section.*
☐	☒	A description of all covariates tested
☐	☒	A description of any assumptions or corrections, such as tests of normality and adjustment for multiple comparisons
☐	☒	A full description of the statistical parameters including central tendency (e.g. means) or other basic estimates (e.g. regression coefficient) AND variation (e.g. standard deviation) or associated estimates of uncertainty (e.g. confidence intervals)
☐	☒	For null hypothesis testing, the test statistic (e.g. F, t, r) with confidence intervals, effect sizes, degrees of freedom and P value noted<br>*Give P values as exact values whenever suitable.*
☐	☒	For Bayesian analysis, information on the choice of priors and Markov chain Monte Carlo settings
☐	☒	For hierarchical and complex designs, identification of the appropriate level for tests and full reporting of outcomes
☐	☒	Estimates of effect sizes (e.g. Cohen's d, Pearson's r), indicating how they were calculated

Our web collection on statistics for biologists contains articles on many of the points above.

Software and code

Policy information about availability of computer code

Data collection	data was re-analyzed from an existing experiment (https://doi.org/10.1038/s41562-019-0801-5)
Data analysis	data were analyzed using python (v3.6.5) [HDDM (v0.9.7), BADS(v1.1.2), and IBS] and custom MATLAB code (v2024a).

For manuscripts utilizing custom algorithms or software that are central to the research but not yet described in published literature, software must be made available to editors and reviewers. We strongly encourage code deposition in a community repository (e.g. GitHub). See the Nature Portfolio guidelines for submitting code & software for further information.

Data

Policy information about availability of data

All manuscripts must include a data availability statement. This statement should provide the following information, where applicable:

- Accession codes, unique identifiers, or web links for publicly available datasets
- A description of any restrictions on data availability
- For clinical datasets or third party data, please ensure that the statement adheres to our policy

data are available from the original authors. Analysis code available at https://github.com/shenhavlab/appearance-of-adaptive-control

Human research participants

Policy information about studies involving human research participants and Sex and Gender in Research.

Reporting on sex and gender	These data were not analyzed in terms of sex or gender. (10 males, 12 females)
Population characteristics	These data were not analyzed in terms of demographics. (19 - 28 years old)
Recruitment	Participants were were recruited by the original experimenters at their university.
Ethics oversight	This projects was approved by the Ethical Committee of Ghent University hospital.

Note that full information on the approval of the study protocol must also be provided in the manuscript.

Field-specific reporting

Please select the one below that is the best fit for your research. If you are not sure, read the appropriate sections before making your selection.

☐ Life sciences ☒ Behavioural & social sciences ☐ Ecological, evolutionary & environmental sciences

For a reference copy of the document with all sections, see nature.com/documents/nr-reporting-summary-flat.pdf

Behavioural & social sciences study design

All studies must disclose on these points even when the disclosure is negative.

Study description	This study is quantitative experiment, re-analyzing an existing dataset (Vassena, E., Deraeve, J. & Alexander, W. H. Surprise, value and control in anterior cingulate cortex during speeded decision-making. Nature Human Behaviour, doi:10.1038/s41562-019-0801-5 (2020).). For full details, refer to the Nature reporting summary published in this original work.
Research sample	The sample was collected by the original authors; consistenting of 'members of the Belgian community associated with Ghent University'
Sampling strategy	The original authors justified their sample size on the basis of previous experiments.
Data collection	We reanalyzed behavior collected during fMRI scanning (3T Magnetom Trio MRI Scanner, Siemens). This experiment was not blinded.
Timing	Data were collected between Jan 13th 2016 and Feb 11 2016.
Data exclusions	One participant was excluded from the original sample due to technical difficulties.
Non-participation	No participants dropped out of the experiment
Randomization	The stimuli were counterbalanced across participants to minimize stimulus-specific confounds.

Reporting for specific materials, systems and methods

We require information from authors about some types of materials, experimental systems and methods used in many studies. Here, indicate whether each material, system or method listed is relevant to your study. If you are not sure if a list item applies to your research, read the appropriate section before selecting a response.

Materials & experimental systems

n/a	Involved in the study	
☒	☐	Antibodies
☒	☐	Eukaryotic cell lines
☒	☐	Palaeontology and archaeology
☒	☐	Animals and other organisms
☒	☐	Clinical data
☒	☐	Dual use research of concern

Methods

n/a	Involved in the study	
☒	☐	ChIP-seq
☒	☐	Flow cytometry
☒	☐	MRI-based neuroimaging
